# Patiëntbetrokkenheid tijdens crisisbesluitvorming: Lessen voor de toekomst op basis van COVID-19

**DOI:** 10.1007/s12508-021-00328-5

**Published:** 2022-01-17

**Authors:** Eduard Schmidt, Suzan van der Pas, Jelmer Schalk, Sietse Wieringa, Britt Kraaijeveld, Sandra Groeneveld, Jet Bussemaker

**Affiliations:** 1 Present Address: Instituut Bestuurskunde, Universiteit Leiden, Leiden, Nederland; 2 Faculteit Sociaal Werk en Toegepaste Psychologie, Hogeschool Leiden, Leiden, Nederland; 3 LUMC-Campus Den Haag, Den Haag, Nederland; 4 LUMC, Leiden, Nederland; 5 Nuffield Department of Primary Care Health Sciences, University of Oxford, Oxford, Verenigd Koninkrijk

## Abstract

Patiëntbetrokkenheid is een belangrijk thema in de zorg. Cliëntenraden vervullen hierin een belangrijke rol. Tegelijkertijd is er nog weinig bekend over hun rol bij de besluitvorming tijdens crises, en wat we van deze ervaringen kunnen leren. De coronacrisis liet echter zien dat wanneer besluitvorming plaatsvindt in interorganisationele netwerken, en sterk top-down en gecentraliseerd is, er weinig ruimte overblijft voor patiëntbetrokkenheid. Wij stellen dat zowel bestuurders als cliëntenraden meer werk moeten maken van patiëntbetrokkenheid in interorganisationele netwerken en tijdens crisissituaties.

## Abstract (en)

Patient involvement is an important topic in health care. Client councils are one way through which patients are involved in decision-making. However, we know little about their role during crises, and what we can learn from these experiences. We argue that there was little patient involvement in the top-down and centralized decision-making during the COVID-19 crisis. Furthermore, many decisions were taken in interorganizational networks, where patient involvement is rare. Based on these findings, we argue that health care organizations and client councils should rethink what effective patient involvement looks like during crises and in interorganizational networks.

## Inleiding

Tijdens de coronacrisis zijn cliëntenraden onvoldoende meegenomen binnen crisisstructuren, hoewel ze een geïnstitutionaliseerd orgaan zijn in zorginstellingen. Betrokkenheid van cliëntenraden had kunnen bijdragen aan de kwaliteit van zorg en legitimiteit van beslissingen doordat ze een aanvullend perspectief bieden. Het belang om patiënten te betrekken bij het reilen en zeilen in de zorg wordt algemeen onderkend [1]. Verschillende belangrijke denkstromen in de zorg, zoals shared decision-making, evidence-based medicine en value-based healthcare benadrukken al jaren het belang van patiëntbetrokkenheid voor de kwaliteit van de besluitvorming en de prestaties van zorgorganisaties [2]. Ook in de bestuurskunde wordt dit belang onderstreept, bijvoorbeeld voor de legitimiteit van besluitvorming [3–5].

De medezeggenschap van patiënten is wettelijk geregeld in de Wet medezeggenschap cliënten zorginstellingen (WMCZ 2018), waarvan de herziening op 1 juli 2020 in werking trad. Deze wet introduceert instemmingsrecht voor cliëntenraden bij een groot aantal onderwerpen die de organisatie van zorginstellingen betreffen, zoals beleid rond kwaliteit, (voedsel)veiligheid en de inrichting van gebouwen bij langdurige zorg. Ook voor cliënten geldende regelingen (zoals de klachten- of bezoekregelingen) en procedures rond het opstellen en bespreken van een zorgplan hebben voor invoering instemming van de cliëntenraad nodig.

De wet trad in werking in een bestuurlijke praktijk die zich kenmerkte door a) hoge druk op de besluitvorming door de coronacrisis en daarmee b) het toenemende belang van interorganisationele samenwerking in de zorg in de regio. De vraag is of en hoe onder deze omstandigheden een grotere inbreng van patiënten werd gerealiseerd.

In dit artikel bekijken we hoe patiëntbetrokkenheid tijdens de coronacrisis werd vormgegeven in een academisch ziekenhuis en binnen een regionaal zorgnetwerk. We stellen dat cliëntenraden niet automatisch een prominente rol binnen crisisstructuren innemen, terwijl ze wel kunnen bijdragen aan de kwaliteit van de zorg en de legitimiteit van beslissingen binnen zorginstellingen. Omdat de coronacrisis eens te meer heeft laten zien dat toenemende samenwerking tussen zorgorganisaties noodzakelijk is, vinden we dat juist in deze context meer nagedacht moet worden over de manier waarop patiëntbetrokkenheid wordt vormgegeven en ingezet.

We staan eerst stil bij onderzoek naar patiëntbetrokkenheid, waarna we inzoomen op patiëntbetrokkenheid tijdens en na crisissituaties. We laten zien waarom er, ondanks de nieuwe wet, nog steeds een aantal belangrijke punten is dat aandacht behoeft en een groot effect heeft op de rol en effectiviteit van cliëntenraden. We doen dat op basis van onderzoek waarbij de impact van de coronacrisis op een academisch ziekenhuis en zorg in de regio is onderzocht [6]. We sluiten af met lessen voor wetenschap en praktijk.

## Onderzoek naar patiëntbetrokkenheid

Verschillende disciplines doen onderzoek naar patiëntbetrokkenheid. Wij concentreren ons op inzichten uit de gezondheidswetenschappen en bestuurskunde. In beide gevallen wordt het belang van de betrokkenheid van burgers bij publieke dienstverlening in het algemeen en van patiënten bij zorgorganisaties in het bijzonder meestal onderbouwd door te wijzen op veranderende ideeën over de rol van burgers in de samenleving. In een samenleving waarin burgers mondiger zijn geworden en waarin ‘actief burgerschap’ wordt verwacht om bij te dragen aan publieke dienstverlening, vormen cliëntenraden een manier om patiëntbetrokkenheid te institutionaliseren [5]. Centrale gedachte hierbij is dat patiëntbetrokkenheid bijdraagt aan de legitimiteit van de besluitvorming in de organisatie en nieuwe perspectieven inbrengt om tot betere besluiten te komen.

Vanuit de gezondheidswetenschappen en geneeskunde is de centrale gedachte dat patiëntbetrokkenheid bij besluitvorming leidt tot betere zorg. Enerzijds omdat deze (meer objectief) de kwaliteit van de informatie verbetert op basis waarvan besluiten worden genomen en anderzijds (meer subjectief) omdat ze tot meer ‘empowerment’ van patiënten en een groter vertrouwen tussen zorgverlener en patiënt leidt [7].

Patiëntbetrokkenheid kan verschillende vormen aannemen. Met *individuele *patiëntbetrokkenheid wordt meestal de stem van de patiënt in het behandelproces bedoeld, waardoor besluiten genomen worden die beter aansluiten bij de behoeften van de patiënt. Het shared decision-making-model werd bij zijn introductie bijvoorbeeld gezien als paradigmaverschuiving, waarbij de relatie tussen arts en patiënt veranderde van een meer autoritair perspectief (de arts beslist) naar een meer gedeelde verantwoordelijkheid [8]. De relatie tussen patiënt en arts heeft dan de vorm van een partnerschap, waarbij patiënten niet louter passieve ontvangers van zorg zijn. Vanuit dezelfde gedachte is er door de jaren heen ook steeds meer aandacht gekomen voor de rol die patiënten als adviseurs van zorgbestuurders kunnen spelen. Cliëntenraden verdiepen zich bijvoorbeeld in het beleid en toetsen dit vanuit het perspectief van de patiënt, om daarmee als *collectief* gemeenschappelijke belangen van patiënten te behartigen. Cliëntenraden bestaan daarbij uit meer dan alleen patiënten, omdat hierin bijvoorbeeld ook familieleden of mantelzorgers van patiënten, ex-patiënten of burgers uit de regio van de zorgorganisatie kunnen plaatsnemen.

In de bestuurskunde is er vooral veel aandacht voor de inbreng van cliëntenraden vanwege de aard van hun rol, namelijk het adviseren van het bestuur van zorginstellingen over de te varen koers, nieuwe initiatieven en de dienstverlening in brede zin. Veel wetenschappelijk werk op dit gebied spreekt hierbij van *coproductie*, wat kan worden gedefinieerd als het leveren van publieke diensten waaraan burgers en professionals samen bijdragen [5]. Onderzoek naar de rol van cliëntenraden laat zien dat patiënten verschillende motieven kunnen hebben om op deze manier bij te dragen aan de dienstverlening van zorginstellingen, maar dat *community motivations* centraal staan [5]. Het gaat hierbij om een bijdrage die verder gaat dan individuele belangen. Onderzoek onder verzorgingstehuizen in de Verenigde Staten laat zien dat een sterkere betrokkenheid van patiënten bijdraagt aan de prestaties van de organisatie [3]. Uit ander bestuurskundig onderzoek blijkt dat coproductie niet alleen het gevoel van zeggenschap over de eigen gezondheid bevordert, maar ook het vertrouwen in zorgprofessionals versterkt [4]. Bedenk dat er veel variatie is in de manier waarop coproductie kan worden vormgegeven. Binnen de zorg wordt vaak gewerkt met de participatieladder, die verschillende vormen van inspraak vanuit patiënten onderscheidt.

## Patiëntbetrokkenheid tijdens de coronacrisis

De coronacrisis zorgt voor een uitvergroting van vraagstukken rond patiëntbetrokkenheid. Juist in crisissituaties, zoals de COVID-19-pandemie, blijkt duidelijk in welke mate het vanzelfsprekend wordt geacht cliëntenraden, en dus het perspectief van de patiënt, bij de besluitvorming te betrekken. In situaties waar bestuurders te maken hebben met een grote mate van onzekerheid en tijdsdruk om snel besluiten te nemen blijkt dat besluitvorming vaak wordt gecentraliseerd in de top van de organisatie [9]. Met andere woorden: besluiten worden meer dan normaal top-down genomen, zonder veel participatie in het besluitvormingsproces. De redenen hiervoor betreffen de onzekerheid, onvoorspelbaarheid, grote dreiging en tijdsdruk waaronder tijdens een crisis snel moet worden gehandeld [10]. In zo’n situatie wordt van bestuurders gevraagd om met beperkte informatie soms verstrekkende besluiten te nemen. Dat levert vaak een duidelijke *command-and-control*-structuur op, die zeker tijdens de eerste golf in maart 2020 te zien was [6]. Zo’n structuur is niet organisch van aard, maar vaak vastgelegd in protocollen en handboeken. In deze crisisorganisatiestructuur neemt een klein team in de organisatie een groot deel van de besluiten, die onder tijdsdruk genomen moeten worden, en communiceert deze naar de rest van de organisatie. De centralisatie van de besluitvorming tijdens crises is niet uniek voor de zorg of de coronacrisis. Ook de besluitvorming van de Rijksoverheid zelf wordt tijdens de coronacrisis als sterk top-down gekenmerkt en ook andere soorten crises (zoals natuurrampen of financiële crises) resulteren vaak in gecentraliseerd bestuur.

Uit onderzoek van Bussemaker et al. in de eerste helft van 2020 in een academisch ziekenhuis bleek dat twee verschillende teams in de crisisstructuur een belangrijke rol speelden [6]. Het crisisteam (CT) was verantwoordelijk voor directe operationele organisatietaken, het crisisbeleidsteam (BT) had de verantwoordelijkheid voor verstrekkende strategische besluiten en de communicatie hierover, zowel intern als extern. Net als bij andere crisissituaties was hier sprake van een duidelijke commandostructuur, waarin via hiërarchische lijnen bepaald wordt wie op welk gebied besluiten neemt [10]. De cliëntenraad van het ziekenhuis was nauwelijks betrokken bij deze crisisteams.

Bij de top-downbesluitvorming in het ziekenhuis bleek (net als landelijk) ook hoe dominant de medisch-curatieve blik was. De combinatie van gecentraliseerde besluitvorming en dominantie van een medisch-curatief perspectief maakte dat er weinig ruimte was voor input van medewerkers en patiënten. Bussemaker et al. [6] laten zien dat dit gedurende langere tijd gold – binnen de crisisorganisatiestructuur werd nauwelijks nagegaan of alle stakeholders daadwerkelijk betrokken werden. Het gevolg daarvan was dat er vooral *voor* in plaats van *met* de patiënt gedacht werd.

## Waar vonden besluiten plaats?

Snelle en top-downbesluitvorming is niet de enige reden dat de patiëntbetrokkenheid minimaal was tijdens de eerste golf van COVID-19-besmettingen. Een andere reden is dat veel, soms verstrekkende besluiten buiten de grenzen van individuele organisaties genomen werden. Als de coronacrisis namelijk iets laat zien, dan is het dat regionale samenwerking tussen organisaties van groot belang is. Al voor de crisis kon geconstateerd worden dat preventie en regionale afstemming tussen zorgorganisaties verbetering behoeven. Tijdens de coronacrisis werd snel duidelijk dat samenwerking in de regio, zelfs tussen concurrenten, noodzakelijk was om de crisis het hoofd te kunnen bieden.

In crisissituaties wordt vaak teruggegrepen op bestaande samenwerkingsstructuren, wat ook tijdens de coronacrisis gebeurde. Bussemaker et al. laten zien dat de omvang en complexiteit van de coronacrisis de behoefte aan kennis- en informatie-uitwisseling vergrootten. De crisis onderstreepte de noodzaak van samenwerking [6]. Vooral het Regionaal Overleg Acute Zorgketen (ROAZ) is een belangrijke netwerkorganisatie waarbinnen verschillende zorgorganisaties elkaar ontmoeten. In toenemende mate werden belangrijke vraagstukken rond de zorg in de regio in dit verband besproken. Sterker nog, de scope van het ROAZ en de intensiteit van de overleggen werden vergroot. Tegelijkertijd is er binnen deze netwerkorganisatie weinig ruimte voor de inbreng van cliëntenraden, laat staan voor geïnstitutionaliseerde patiëntbetrokkenheid. Juist omdat cliëntenraden tot één organisatie behoren, is er binnen het ROAZ niet direct een plek waar patiënten van verschillende organisaties kunnen adviseren en bijdragen aan de besluitvorming. Daar komt bij dat er tijdens crises vaak geen continue reflectie is op de vraag of de juiste stakeholders aan tafel zitten en of de contacten met hun achterban goed geregeld zijn. In veel gevallen werden de *buiten* de grenzen van de zorgorganisatie genomen besluiten onvoldoende gestaafd aan de gevolgen voor de patiënt *binnen* de zorgorganisatie.

## Lessen uit de coronacrisis

Een belangrijke conclusie over de COVID-19-respons van zorgorganisaties is dat cliëntenraden, ondanks een geïnstitutionaliseerde positie in de organisatiestructuur, niet automatisch een prominente rol binnen crisisstructuren krijgen. Dit terwijl het perspectief van patiënten potentieel bijdraagt aan de kwaliteit van de zorg en de legitimiteit van en steun voor de besluitvorming en beslissingen binnen zorginstellingen, ook tijdens crises.

Met het toenemende belang van interorganisationele samenwerking in het achterhoofd is het belangrijk om na te denken over de manier waarop patiëntbetrokkenheid ook in deze netwerken kan worden vormgegeven. Zo zouden vanuit verschillende cliëntenraden liaisons met cliëntenraden in andere zorgorganisaties in de regio kunnen worden aangegaan, naast de contacten die ze al hebben met vergelijkbare organisaties buiten de regio (zoals cliëntenraden van academische centra onderling). Dat gaat niet vanzelf. Een rol binnen interorganisationele samenwerkingsverbanden past op dit moment niet bij de wettelijke taak van cliëntenraden en roept de principiële vraag op of cliëntenraden er zijn voor de eigen zorginstelling of voor een hoger doel: betere zorg voor alle burgers in de regio. Ook in praktisch opzicht kan dit nieuwe uitdagingen opleveren, bijvoorbeeld wanneer een cliëntenraad binnen een regionaal samenwerkingsverband iets adviseert wat voor de eigen zorginstelling niet de beste uitkomst is. Het is de vraag hoe het cliëntenperspectief binnen samenwerkingsverbanden het beste kan worden georganiseerd.

Daarnaast is het van belang om stil te staan bij de wijze waarop invulling kan worden gegeven aan het betrekken van cliëntenraden in een situatie waarbij snelle besluitvorming is vereist. Hierbij dient er niet halsstarrig te worden vastgehouden aan één (crisis)besluitvormingsstructuur. Besluitvorming in een meer top-downbestuur moet worden afgewisseld met besluitvorming waarin meer ruimte is voor reflectie en tegengeluiden. Belangrijk is dat bestuurders zich realiseren dat een specifieke crisisorganisatiestructuur niet op elk moment van een crisis passend is. Tijdens de crisis horen cliëntenraden uit de eerste hand welke effecten crisismaatregelen in het ziekenhuis hebben. Ze zien niet alleen waar zorgprofessionals tegenaan lopen, maar ook waar patiënten moeite mee hebben of wat niet goed gaat. Ze kunnen inzicht geven in de manier waarop patiënten lastige dilemma’s ervaren. Een voorbeeld hiervan is het afwegen van het beperken van bezoekregelingen ten behoeve van de veiligheid in het ziekenhuis tegen het effect van zo’n maatregel op het welzijn van patiënten. Juist cliëntenraden kunnen helpen om te reflecteren op de keuzen bij dit soort lastige vraagstukken, en het beleid dat uiteindelijk gekozen is kan door hun bijdrage mogelijk ook op meer draagvlak en legitimiteit rekenen. Het kan al helpen om de samenwerking tussen bestuurders en cliëntenraad te versterken wanneer bestuurders en cliëntenraad elkaar informeel opzoeken om te sparren over de uitdagingen waarvoor de organisatie staat. Door vroegtijdig de blik van patiënten en cliënten serieus te nemen, en hen continu te vragen om op de genomen besluiten te reflecteren, worden bestuurders scherp gehouden en kan er snel van koers gewisseld worden, mocht dat nodig zijn.

Ook cliëntenraden zelf kunnen leren van hun rol tijdens de eerste golf van COVID-19-besmettingen. Ze kunnen bijvoorbeeld zelf actief op zoek gaan naar de goede initiatieven die tijdens crisissituaties naar boven zijn gekomen, en helpen om deze te verspreiden en zo nodig te institutionaliseren in de organisatie. Veel goede initiatieven die tijdens de coronacrisis zijn ontstaan, komen niet voorbij de grenzen van de afdeling waar ze zijn ontwikkeld. Daarnaast kan onderling contact tussen cliëntenraden van verschillende instellingen een rol spelen bij het naar binnen brengen van nieuwe perspectieven en ideeën, waardoor het bestuur scherp wordt gehouden. Mede vanwege hun ervaringen met beleid kunnen cliëntenraden een belangrijke rol spelen bij het aanpassen van crisisprotocollen, bijvoorbeeld als het gaat om de manier waarop over nieuwe regels gecommuniceerd wordt en op welke wijze bezoekers van zorgorganisaties aangespoord kunnen worden om continu veranderende (gedrags)regels in acht te nemen en te accepteren.

## Conclusie

Het betrekken van de patiënt bij besluitvorming is nog steeds niet de standaard in de organisatie van de zorg, ondanks het grote belang dat eraan wordt gehecht. Juist in crisissituaties wordt dit pijnlijk voelbaar, omdat patiëntbetrokkenheid nog makkelijker uit het oog wordt verloren door de grote druk op zorgorganisaties en zorgbestuurders. Het is dus niet verwonderlijk, maar wel zorgelijk, dat de cliëntenraden gedurende de coronacrisis niet automatisch een prominente plaats in het besluitvormingsproces innamen. Het managen van crisissituaties vraagt om bestuur dat kan balanceren tussen snelle, top-downbesluitvorming als de situatie daarom vraagt, en meer participatieve vormen van besluitvorming zodra de omstandigheden dat toelaten. Die participatie moet, ook in de standaard crisisprotocollen, vorm krijgen in de organisatie en ingebed zijn in de mindset van bestuurders, voordat een crisis uitbreekt. Dat betekent dat ook cliëntenraden zelf meer moeten nadenken over de rol die ze willen spelen tijdens een crisis en meer samenwerking moeten zoeken met cliëntenraden van andersoortige zorginstellingen in de regio.

## References

[1] Teunissen GJ, Abma TA (2010). Derde partij: tussen droom en daad. Tijdschr Gezondheidswet.

[2] Porter ME, Teisberg EO (2006). Redefining health care: creating value-based competition on results.

[3] Amirkhanyan AA, Cheon O, Davis JA (2019). Citizen participation and its impact on performance in U.S. nursing homes. Am Rev Public Adm.

[4] Jo S, Nabatchi T (2019). Coproducing healthcare: individual-level impacts of engaging citizens to develop recommendations for reducing diagnostic error. Public Manag Rev.

[5] van Eijk CJA, Steen TPS (2014). Why people co-produce: analysing citizens’ perceptions on co-planning engagement in health care services. Public Manag Rev.

[6] Bussemaker M, Groeneveld SM, Wieringa S (2021). COVID-crisis of COVID-kans? Adaptief en lerend bestuur in het LUMC en de regio.

[7] de Wit M, Bloemkolk D, Teunissen T (2016). Voorwaarden voor succesvolle betrokkenheid van patiënten/cliënten bij medisch wetenschappelijk onderzoek. Tijdschr Gezondheidswet.

[8] Goossensen A, Zijlstra P (2007). Shared decision making in de psychiatrie. Tijdschr Gezondheidswet.

[9] Boin A, Hart P (2003). Public leadership in times of crisis: mission impossible?. Public Adm Rev.

[10] Moynihan DP (2008). Learning under uncertainty: networks in crisis management. Public Adm Rev.

